# Supplementary material for: Mechanisms governing the pioneering and redistribution capabilities of the non-classical pioneer PU.1
Source: Nat Commun. 2020 Jan 21;11:402. doi: 10.1038/s41467-019-13960-2 (PMC6972792; doi:10.1038/s41467-019-13960-2)
Supplement: Supplementary file 7 — Source data [file 41467_2019_13960_MOESM7_ESM.zip › Source_Data/Figure6/Figure6B_MotifScanOutput/ETS1_PU.1/homerResults/motif27.similar.html]

motif27

## Information for motif27

C
T
A
G
A
C
T
G
A
C
T
G
G
A
T
C
A
C
T
G
A
C
T
G
C
T
A
G
T
C
A
G
A
G
T
C
  
Reverse Opposite:  

C
T
A
G
A
G
T
C
A
G
T
C
G
T
A
C
A
G
T
C
C
T
A
G
A
G
T
C
A
G
T
C
G
A
T
C
  

|  |  |
| --- | --- |
| p-value: | 1e-71 |
| log p-value: | -1.637e+02 |
| Information Content per bp: | 1.845 |
| Number of Target Sequences with motif | 1523.0 |
| Percentage of Target Sequences with motif | 9.62% |
| Number of Background Sequences with motif | 1660.7 |
| Percentage of Background Sequences with motif | 5.18% |
| Average Position of motif in Targets | 100.5 +/- 57.7bp |
| Average Position of motif in Background | 91.5 +/- 65.4bp |
| Strand Bias (log2 ratio + to - strand density) | 0.1 |
| Multiplicity (# of sites on avg that occur together) | 1.17 |
| Motif File: | file (matrix) reverse opposite |

### Similar de novo motifs found

|  |  |  |  |  |  |  |  |
| --- | --- | --- | --- | --- | --- | --- | --- |
| Rank | Match Score | Redundant Motif | P-value | log P-value | % of Targets | % of Background | Motif file |
| 1 | 0.953 | C T A G A C T G A C T G G A T C A C T G A C T G C T A G T A C G A G T C A G T C G C T A | 1e-70 | -161.656298 | 6.65% | 3.05% | motif file (matrix) |
| 2 | 0.955 | T G C A G C A T C T A G T C A G A T G C A G T C T G A C G T A C C T A G A G T C A T G C G A T C | 1e-62 | -142.877669 | 8.96% | 4.93% | motif file (matrix) |
| 3 | 0.896 | G C A T C T A G C T A G A G T C A G T C T G A C T A G C T C A G G T A C A T G C | 1e-50 | -117.280001 | 8.14% | 4.63% | motif file (matrix) |
